# Metabarcoding data of bacterial diversity of the deep sea shark, *Centroscyllium fabricii*

**DOI:** 10.1016/j.dib.2018.10.062

**Published:** 2018-10-24

**Authors:** Tina Kollannoor Johny, Bindiya Ellathuparambil Saidumohamed, Raghul Subin Sasidharan, Sarita Ganapathy Bhat

**Affiliations:** Department of Biotechnology, Cochin University of Science and Technology, Kalamassery, Cochin 682022, Kerala, India

**Keywords:** *Centroscyllium fabricii*, Gut microbiota, Metagenome, Illumina

## Abstract

This data article describes the bacterial diversity of the deep sea shark, *Centroscyllium fabricii*. The data was acquired by metabarcoding using 16S rDNA. *Centroscyllium fabricii*, a deep sea shark found at depths below 275 m was sampled during Sagar Sampada cruise no 305 in the Indian Ocean and metagenomic DNA was isolated from the gut contents using QIAamp DNA stool minikit. V3 region of 16S rDNA region was amplified and the amplicons were sequenced on Illumina MiSeq system using 151 bp × 2 paired end reads. The data of this metagenome is available in the BioSample Submission Portal as Bio-Project PRJNA431407and Sequence Read Archive (SRA) accession number SRR6507004.

**Specifications table**

**Table:** 

Subject area	*Biology*
More specific subject area	*Metagenomics*
Type of data	*FastQ file*
How data was acquired	*Illumina MiSeq*
Data format	*Raw*
Experimental factors	*Environmental sample*
Experimental features	*Metagenomic DNA extraction and sequencing of V3 region of 16S rDNA*
Data source location	*Arabian Sea, India (8° 11.4” N and 75° 54.9” E)*
Data accessibility	*The data of this metagenome is available in the NCBI BioSample Submission Portal as Bioproject PRJNA431407and SRA accession number* SRR6507004

**Value of the data**

• This is the first data report on bacterial diversity of deep sea fish gut from the Oxygen Minimum zone of Indian Ocean.
• Generation of an inventory of bacterial diversity of *Centroscyllium fabricii* gut can be regarded as a first step towards recognition of its bioactive potential.
• The significant proportion of hitherto unstudied bacterial phyla (25.30%) identified in the dataset indicates immense scope for Blue Biotechnology.
• The data provided here can also be used to understand host-microbiome interactions and to shed light on the factors that influence the establishment of gut microbiota.

## 1 Data

With 33,700 species described to date [1], fishes are important benefactors of the exceptional marine biodiversity spanning various hierarchical levels. Fishes harbor bacterial populations on almost every organ [2], however, the colonization of the gut environment is the most intricate process, governed by external factors and other selective pressures within [3]. *Centroscyllium fabricii* are deep water schooling sharks found at depths ranging from 180–2250 m [4]. This zone of the ocean is characterized by low nutrient availability, high salt, low temperature and high pressure [5] These extreme conditions contribute to the evolution of diverse adaptations in the animal microbiomes [6]. As most of the marine microbes are uncultivable [7], only a metagenomic approach can be used to gain a comprehensive understanding of the community composition and function of the microbiome.

**Fig. 1:**
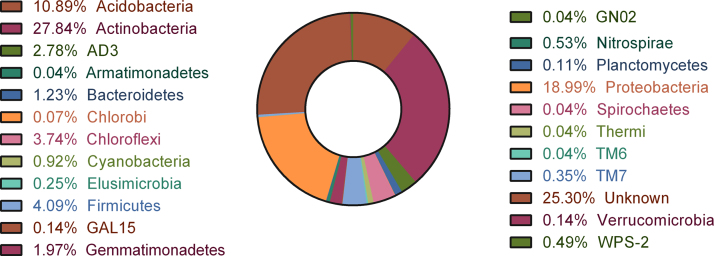
Taxonomic classification of OTUs at phylum level for the *Centroscyllium fabricii* gut sample.

**Fig. 2:**
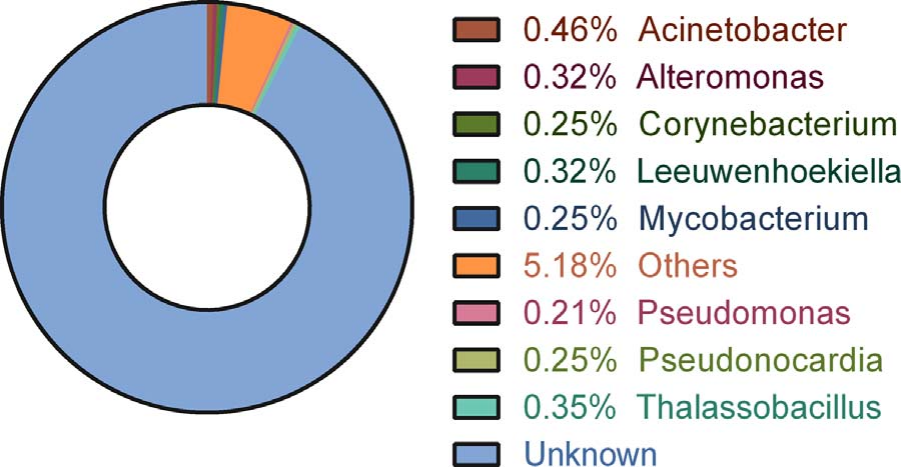
Taxonomic classification of OTUs at genus level for the *Centroscyllium fabricii* gut sample. Only the top 10 genera are summarized here.

DNA metabarcoding centered on the 16S rDNA sequence is a high-throughput approach used to catalog taxonomic diversity of environmental samples. The data presented here was obtained by Illumina MiSeq sequencing of V3 region of 16SrDNA. A total of 23 bacterial phyla were identified in the dataset, as shown in Fig. 1. The dominant phyla in this microenvironment were *Actinobacteria* (27.84%), *Proteobacteria* (18.99%) and *Acidobacteria* (10.89%). 25.30% of Operational Taxonomic Units (OTUs) did not have any significant hits against the taxonomic database and was categorized as unknown. 107 genera were identified in the dataset, but the abundance of each was found to be less than 1%. *Acenitobacter* (0.46%) was predominant at the genus level of taxonomic resolution. A vast majority of OTUs (92.4%) remained unclassified at this level. The top 10 genera including unknown are depicted in Fig. 2. The complete list of genera identified in the data is given in Supplementary file 1.

## 2 Experimental design, materials and methods

### 2.1 Sample collection

The deep sea shark, *Centroscyllium fabricii* was sampled onboard the Fishery Oceanographic Research Vessel (FORV) SagarSampada cruise #305 in the Indian Ocean by using HSDT (High Speed Demersal Trawl) net (8° 11.4” N and 75° 54.9” E). The fish was subjected to molecular identification by DNA isolation and PCR amplification of 5’ region of cytochrome c oxidase subunit I (cox1) gene from mitochondrial DNA using universal fish primers F2 and R2 [8]. The amplicons were sequenced and subjected to BLAST analysis. The sequences were submitted to GenBank and accession number was obtained (KT905423.1).

### 2.2 DNA extraction and metagenome sequencing

Metagenomic DNA was isolated from the gut contents using QIAamp DNA stool minikit (Qiagen, India) and the hypervariable V3 region of 16S rRNA gene was amplified using primers 341F 5’CCTACGGGAGGCAGCAG 3’ and 518R 5’ATTACCGCGGCTGCTGG 3’. The amplicons were sequenced on Illumina MiSeq system using 151 bp × 2 paired end reads. The raw sequence files were analyzed for base quality, base composition and GC content. V3 region was extracted from paired end reads by trimming of spacer and conserved region and by building a consensus V3 region from the trimmed paired end reads. High quality V3 sequences were extracted by passing the reads through filters for spacer region, conserved region, read quality and mismatch. Subsequent analyses were performed using Quantitative Insights Into Microbial Ecology (QIIME) pipeline.

## Appendix A Supplementary material

**Supplementary file 1.** Complete list of OTUs at genus level for the *Centroscyllium fabricii* gut sample. Supplementary material
