# Supplementary material for: Metabarcoding data of bacterial diversity of the deep sea shark, Centroscyllium fabricii
Source: Data Brief. 2018 Oct 24;21:1029–32. doi: 10.1016/j.dib.2018.10.062 (PMC6226572; doi:10.1016/j.dib.2018.10.062)
Supplement: Supplementary file 1 — Transparency document [file mmc1.pdf]

CONFLICT OF INTEREST FORM

The authors declare that there are no known conflicts of interest associated with the data presented here and there has been no significant financial support for this work that could have influenced its outcome.

Signed by all authors as follows:

1	Tina Kollannoor Johny	[illegible] 21/2/18
2	Bindiya Ellathuparambil Saidumohamed	Bindiya 21/2/18
3	Dr. Raghul Subin Sasidharan	Raghul 21-02-2018
4	Dr. Sarita Ganapathy Bhat<br>(Corresponding author)	[illegible] 21/2/18
